# ‘*I can see what's going on without being nosey…*’: What matters to people living with dementia about home as revealed through visual home tours

**DOI:** 10.1002/gps.5999

**Published:** 2023-09-08

**Authors:** Sarah Campbell, Andrew Clark, John Keady, Kainde Manji, Elzana Odzakovic, Kirstein Rummery, Richard Ward

**Affiliations:** ^1^ Manchester Metropolitan University Manchester UK; ^2^ University of Salford Salford UK; ^3^ University of Manchester Manchester UK; ^4^ Age Scotland Scotland UK; ^5^ Jönköping University Jonkoping UK; ^6^ University of Stirling Stirling UK

**Keywords:** ageing in place, connections, dementia, display, everyday routines, gardens and outdoor spaces, home tours, home, neighbourhood, participatory, visual data

## Abstract

**Objectives:**

This paper considers home from the perspective of people living with dementia supporting ongoing discourse around ageing in place and the significance of creating more inclusive communities.

**Methods:**

Forty‐six home tour interviews led by people living with dementia were conducted in England and Scotland to better understand the connectivity between home and neighbourhood for people living with dementia. These interviews used a range of participatory and creative approaches including video, photographic images and in situ interviews. Data were analysed via reflexive thematic analysis.

**Results:**

Three themes were identified in data analysis. 1. Connected home and neighbourhood, where participants revealed the dynamic relationship between home and neighbourhood; 2. Practices of home, where participants discussed the everyday nature of their homes and routines; and 3. Displaying home and family, which reflected participant's biographical homes in the context of living with dementia.

**Discussion:**

The findings show that home holds multiple meanings for people living with dementia. For example, home is understood as a part of the neighbourhood and an extension of the home space into gardens and backyards, thus extending existing discourses that solely focus on the inside of people's homes. For people living with dementia, homes are also sites of negotiation and renegotiation where new meanings are created to reflect the changing nature and context of the home. There is not one fixed solution to these issues. Support and understanding for people living with dementia will need to evolve to adapt to the shifting dynamics and multiple meanings of home.

## Key points

- Neighbourhoods begin within homes and are interconnected. This relationship has important implications for the discourse of ageing in place.
- ‘Home’ for people living with dementia holds the same meanings for those without dementia, but with some notable differences. It is important to recognise the shifting dynamics of home and to recognise the complexity of home in the context of dementia. There is not a fixed solution to the challenges of ageing at home with dementia, but support and understanding need to evolve alongside people with lived experience.
- Homes are a site of renegotiation where new meanings are created to reflect the changing nature of home and the lived experience of home for those with dementia.
- The study has provided findings through innovation in the research design. The employment of creative methods enabled people living with dementia to participate in sharing their own narratives of home.
- The work provides evidence where there is a current gap in understanding ‘what matters’ about home for people living with dementia.

## 1 BACKGROUND

Dementia is a global issue with an estimated 50 billion people living with dementia across the world, a figure set to rise to 152 billion by 2050. In the United Kingdom (UK) there are predicted to be 944,000 people living with dementia.^1^ Importantly, most people living with dementia (61%) are living at home in their neighbourhoods and communities, some alone and others in the context of a relationship.^1^ The progressive nature of the condition means that, over time, those living with dementia may face challenges in their everyday life, such as difficulty in maintaining social relationships, getting out and about, and continuing to engage in the activities that the person had prior to the onset of dementia.^2^ This means that it is important to understand the experience and meaning of home for people living with dementia to ensure that they are supported in the most meaningful way. Moreover, and as wider discourses about ageing in place also attest, it is valuable to assert the rights of people living with dementia to continue to live their lives within their homes, neighbourhoods and wider communities.^3^

To date, considerations of ‘home’ have received much attention in the sociological literature. For example, ‘home’ has been considered as: the site where everyday life plays out^4^, ^5^, ^6^, ^7^, ^8^; the location where family life and intimate personal relationships are undertaken^9^, ^10^ places where identity can be displayed, shaped and reformed^6^, ^9^, ^10^; a space for politicised and gendered activity that is best understood through the routines and activities of everyday life^11^, ^12^, ^13^; and a place of belonging.^7^, ^14^ At the other end of the spectrum, the home can also be seen as a place of danger and disconnection.^11^ Moreover, ‘home’ has also has been explored in relation to the experience of growing older through the construct of ageing in place where attention is focused on familiarity and connections.^15^

In dementia studies, Cahill^16^ seeks to bring a rights‐based approach to dementia that argues that a person, no matter their capacity, has the right live out their life in their home and their communities. Whilst Bartlett and Brannelly^2^ have taken a theoretical and broad‐based perspective and combined social citizenship with an ethic of care to argue for a more nuanced understanding of life at home for people living with dementia. In particular, Bartlett and Brannelly^2^ wanted to better understand the potential for people with dementia to live well at home by ‘balancing independence, risk, and freedom in the face of increased dependency’ (p.78). This is an important point as home for people living with dementia may also become a space that is no longer private, but one where the person is constantly under surveillance and scrutiny.^2^ As such, home and family life can take on new meanings and identities, and not always for the better.

Illustrative of this more nuanced understanding, Nygård^17^ explored life at home for people with dementia who lived alone and investigated the person's interactions with everyday objects and technologies, such as televisions, kettles, and cookers. These interactions were seen to be both creative and challenging in equal measure and where improvement in person‐centred design for people living with dementia would help to accommodate changing skills and abilities.^18^, ^19^ In addition, Newton et al's.^18^ systematic narrative review on care home and domestic gardens and the experience of living with dementia, revealed the garden as a neglected area of attention. This was mainly due to existing studies on the home failing to accommodate outside spaces of the home within their study design and operational definitions. Other authors have focussed on the home becoming a ‘contested’ site of everyday life where people living with dementia may become increasingly unable to successfully undertake everyday domestic activities, such as cooking, cleaning, or shopping.^20^, ^21^ This steady withdrawal of domestic labour can lead to friction and tension in households when roles and abilities have to be constantly negotiated, renegotiated and accommodated.^20^, ^21^

Mirroring the work of Nygård,^17^ De Witt^22^ revealed the ‘emotional dread’ that women who live alone with dementia experience when considering their future, and the prospect of having to leave their home to move to a supported facility at an unknown future time. In contrast, Koo et al.^23^ explored intergenerational relationships within Singapore‐Chinese families affected by dementia and highlighted the importance of connections between family members and how these relationships are key to the well‐being of life at home.

Home, in the context of the lived experience of dementia, has therefore tended to be considered in three broad areas; (i) to the challenges that need to be met in the on‐going progression of dementia; (ii) in relation to the care needs of the person living with dementia; and (iii) in considering the impact of dementia on relationships within the home. To date, home has not been considered with reference to what people living with dementia continue to do there, or with regards to the relationship between home and neighbourhood. This kind of exploration is crucial in the discourse of empowerment,^24^ rights^3^, ^25^ and citizenship^2^ and supports opportunities to challenge stigma^25^ experienced by people living with dementia as everyday citizens within their homes and neighbourhoods. It is at this nexus where this article, and the reported study, is located.

## 2 METHODS

### 2.1 Study setting

This work was undertaken as part of the Neighbourhoods: Our People, Our Places (N:OPOP) study which was nested within a larger, five‐year programme of work with a focus on Neighbourhoods and dementia (see: https://sites.manchester.ac.uk/neighbourhoods‐and‐dementia/; accessed 20 March 2023). The N:OPOP study set out to explore how people living with dementia experienced their neighbourhoods, and to better understand how neighbourhoods and local communities supported people living with dementia to remain socially and physically active. Central to the N:OPOP study was the aim to work alongside people living with dementia and to find approaches to the research design that involved people in more inclusive ways.^26^ Participatory research has been described as more of ‘an attitude or approach than a series of techniques’^27^ and our approach has been to be inclusive and flexible throughout the research process. Early in the development of the project, we worked with dementia campaign and advocacy groups to shape data collection, analysis and dissemination techniques.^28^ We conducted pilot interviews with advisors from the dementia campaign and advocacy groups to trial the methods, which were designed to be flexible and to enable people living with dementia to lead the data collection. For example, with the home tours, participants could choose to use video camera or photography as we moved around their homes, they could determine the length of interview and what and where they showed us within their homes, gardens and neighbourhoods.^29^ These methods proved to be both popular and enjoyable for participants.

The N:OPOP team were influenced by the work of sensory ethnographer Sarah Pink who established the idea of the ‘sensory home’ through the use of video home tours, and who noted the importance of the method in constructing both visual and verbal representations of everyday life at home.^12^, ^13^ Pink first used ‘home tours’ in her work exploring gender and domestic home life^13^ and, more recently, exploring energy use in different family contexts.^12^ Hence, the method has been developed for use to explore everyday practices of home, and to engage with the ‘feel’ of home. These kind of multi‐sensory methods have been an extremely useful tool for research in the context of dementia because they do not prioritise verbal language in data gathering.^30^ Moreover, the methods proved particularly useful in the context of the lived experience of dementia for their flexible and adaptive qualities.

Within dementia research, ‘in‐situ’ interviews and visual methods have been found to be empowering for participants as methods that allow people living with dementia to be prompted by what is around them, rather than having to ‘think back’ to a memory or remember a space accurately.^31^ Movement through participants' homes using the home tour method was an important way to provide insight into how people living with dementia made sense of their surroundings and engaged with their homes.^32^ In the event, and as we will shortly address, the home tours revealed the fluidity of home and showed that homes stretched beyond their boundary walls into gardens, backyards, and balconies. Homes and neighbourhoods also flowed in and out from each other through windows and over fences thereby creating new threshold spaces.^33^, ^34^

### 2.2 Participants, recruitment and ethics

This paper reports on 46 participatory home tours led by people living with dementia. The tours were carried out in 2015 and 2016 within the UK field sites of the N:OPOP study, namely: Greater Manchester, England and The Forth Valley, Scotland (see Table 1). Of the 46 home tours, 36 were video home tours, 10 were audio tours with photography.

**TABLE 1:** Participants table.

Participant name (pseudonym)	Fieldsite	Gender	Age	Living situation	Type of house/neighbourhood	Length of time in house and neighbourhood	Video or audio with photography tour
Albert	Greater Manchester, England	M	79	Living with spouse	Terrace house—urban	47 years	VIDEO
Sandra	Greater Manchester, England	F	60	Living with spouse	Sheltered high‐rise flat—urban	6 months	VIDEO
Douglas	Greater Manchester, England	M	73	Living with granddaughter	Terrace house—urban	30 years	AUDIO/PHOTOGRAPHY
Amobi	Greater Manchester, England	M	76	Living with spouse	Semi‐detached house—urban	42 years	VIDEO
Florrie	Greater Manchester, England	F	72	Living with spouse	Semi‐detached house—urban	36 years	VIDEO
Suzanne	Greater Manchester, England	F	62	Living with spouse	Detached house—suburban	22 years	VIDEO
Susan	Greater Manchester, England	F	57	Living with spouse	Terrace house ‐ urban	3.5 years	VIDEO
Jean	Greater Manchester, England	F	63	Living with spouse	Terrace house ‐ urban	43 years	VIDEO
Celia	Greater Manchester, England	F	72	Living with spouse	Terrace house ‐ urban	52 years	VIDEO
Sal	Greater Manchester, England	F	67	Living with spouse	Semi‐detached house—urban	21 years	VIDEO (phase 2): CAMERA FAILED IN PHASE 1
Desmond	Greater Manchester, England	M	76	Living with spouse	Ground floor flat ‐ urban	2 months	VIDEO
Robert	Greater Manchester, England	M	80	Living with spouse	Semi‐detached house ‐ urban	40 years	VIDEO
Adam	Greater Manchester, England	M	75	Living with spouse	Semi‐detached house ‐ urban	4 years	VIDEO
Dennis	Greater Manchester, England	M	64	Living with spouse	Semi‐detached house ‐ suburban	27 years	VIDEO
Katherine	Greater Manchester, England	F	76	Living with spouse	Detached house—urban	30 years	AUDIO/PHOTOGRAPHY
Vanessa	Greater Manchester, England	F	61	Living with daughter	Semi‐detached house—urban	21 years	AUDIO/PHOTOGRAPHY
Roger	Greater Manchester, England	M	77	Living with spouse	Semi‐detached house—urban	5.5 years	VIDEO
Phillip	Greater Manchester, England	M	86	Living with spouse	Semi‐detached house—urban	60 years	VIDEO
Isobel	Greater Manchester, England	F	80	Living with spouse	Detached house—suburban	12 years	VIDEO
Thomas	Greater Manchester, England	M	84	Living with spouse	Semi‐detached house ‐ urban	7 years	VIDEO
Kate	Greater Manchester, England	F	89	Living with spouse	Semi‐detached bungalow—urban	45 years	VIDEO
Lily	Greater Manchester, England	F	80	Living alone	Sheltered two‐room low‐rise flat—urban	2 years	VIDEO
Grace	Greater Manchester, England	F	77	Living alone	Sheltered two‐room low‐rise flat—urban	7 months	VIDEO
Dylan	Greater Manchester, England	M	68	Living with spouse	Detached house—suburban	2 years	VIDEO
Margaret	Greater Manchester, England	F	80	Living alone	Semi‐detached house—urban	30+ years	VIDEO
Mike	Greater Manchester, England	M	67	Living alone	High rise—top floor flat—urban	49 years	VIDEO
Anna	Greater Manchester, England	F	D/K	Living alone	Sheltered two‐room low‐rise flat—suburban	6 months	VIDEO
Bob	Forth Valley, Scotland	M	65	Living with spouse	Terrace house ‐ Semi‐rural village	(Not stated but born in village)	VIDEO
John	Forth Valley, Scotland	M	75	Living with spouse	Terrace house—Small town	30 years	AUDIO/PHOTOGRAPHY
Eric	Forth Valley, Scotland	M	75	Living with spouse	First floor flat—urban	Under a year	VIDEO (phase 2)
June	Forth Valley, Scotland	F	79	Living alone	Bungalow, small town	40+ years	AUDIO/PHOTOGRAPHY
Judy	Forth Valley, Scotland	F	67	Living with spouse	Detached house—Small town	9 years	VIDEO (phase 2)
Peter	Forth Valley, Scotland	M	D/K	Living with spouse	Terrace house—Semi‐rural	42 years	AUDIO/PHOTOGRAPHY
Mac	Forth Valley, Scotland	M	79	Living with spouse	Terrace—Village	30 years	AUDIO/PHOTOGRAPHY
Harrold	Forth Valley, Scotland	M	D/K	Living alone	Semi‐detached cottage, farm, rural	12–14 years (location whole life)	VIDEO
James	Forth Valley, Scotland	M	86	Living with spouse	Detached house, Village	At least 30 years	AUDIO/PHOTOGRAPHY
Claire	Forth Valley, Scotland	F	D/K	Living with spouse	Detached house, Village	28 years	VIDEO
Peggy	Forth Valley, Scotland	F	88	Living alone	Flat, Suburban	20 years (approx)	VIDEO
Sean	Forth Valley, Scotland	M	d/k	Living with spouse	Terrace, Urban	38 years	VIDEO
Alec	Forth Valley, Scotland	M	68	Living with spouse	Detached house, rural town	14 years	VIDEO
Andrea	Forth Valley, Scotland	F	d/k	Living with spouse	Terraced house, Urban	1 year	AUDIO/PHOTOGRAPHY
Brenden	Forth Valley, Scotland	M	82	Living with spouse	Detached house, rural town	40 years	VIDEO
Murray	Forth Valley, Scotland	M	59	Living with spouse	Flat, Urban	1.5 years	VIDEO
George	Forth Valley, Scotland	M	76	Living alone	High rise flat, Urban	14—15 years	VIDEO
Kathleen	Forth Valley, Scotland	F	86	Living alone	Flat, Urban	30 years	AUDIO/PHOTOGRAPHY
Doug	Forth Valley, Scotland	M	56	Living with spouse	Detached house, Urban	10 years	VIDEO

Home tours are part of a wider collection of ‘elicitation techniques’ used in the social sciences and the rooms we moved through and the objects we were shown and observed directed and informed the discussions we had with participants. Participants were prompted with questions about what the room in the house was used for, and what was important to them in the space, including outside spaces. Ethical approval to conduct the study was obtained via the applicable ethical governance systems in each locality, including the NHS Health and Social Care panel (REC reference: 15/IEC08/0007).

Obtaining ethical approval took time and resource to develop a set of accessible participant information and consent processes. As the research team were using visual methods with participants in their homes, we developed an ethical protocol setting out how the video and photographic data would be used. This set out how confidentiality and anonymity would be maintained. We included in the ethics application that we might share video or photographs to illustrate aspects of home life, but this would be done without identifying a person or their home location. Further to this, we considered how we would transfer video and photographic images from our equipment to our university storage systems.

We aimed to develop relationships of trust with participants, to ensure that they felt comfortable with us in their homes.^35^ We negotiated consent throughout the process of the home tour, reminding participants that they should only share spaces they felt comfortable to do so. We were very aware that some aspects of home felt like private spaces, not usually shared with strangers. Some participants did opt not to be video recorded although they were comfortable with photographs of their home being taken.

Within our ethics application we discussed capacity issues, and at the first set of interviews participants were required to have capacity to provide informed consent.^36^ Most of the home tours were undertaken during the first phase of data collection. However, a small number (*n* = 3) were undertaken in the second phase of data collection, only one of these participants was no longer able to give informed consent for themselves. As such where a participant no longer had capacity to take part a personal consultee was asked for assent.^36^ We established in the first phase of data collection who the personal consultee would be should they be required. Consent processes with participants were multi‐layered and included providing enough time to discuss the information, the aims of the project and the methods we were proposing to adopt, as well as informing participants what we planned to use the data for. We ensured participants were aware that they could stop at any time and pause, or even stop being involved with the project. Participants were asked if they could tell us about why they wanted to be involved in the project to help us establish they were able to give their informed consent. Participants were also given the option of signing a consent form or verbally recording their consent. We also employed a process consent approach^37^ as we would be visiting participants on a number of occasions through the life of the project. It was therefore important that we renegotiated consent at the time of each visit, and sometimes during sessions too if required.^38^

Ahead of the home tours, participants were told the types of questions they would be asked such as showing and telling the researchers about the spaces and objects in the home that held meaning to them. All the home tours in the study were led by the person living with dementia and the designated researcher either followed behind with a camcorder, or audio recorder and camera, and asked questions as movements were made through the home. As seen in Table 1, 25 participants were men and 21 were women, and they ranged in age from 56 to 89 years old; and they lived in different types of homes and locations. The home tours ranged in length from 13 to 59 min. All names used in the article are pseudonyms.

### 2.3 Data analysis

Data generation and analysis required both a collaborative and reflexive approach to consider the aspects of home that were shown on the tour.^39^ The data analysis was carried out manually. Each video was watched repeatedly by one researcher (SC) and each transcript was read in full several times. The accompanying photographs were viewed alongside the audio. A selection of videos were watched by two of the other authors (JK and AC) and discussed with SC to reflect on the analysis and to enable discussion and reflexivity of the analytical categories.^39^ A set of open codes were developed from the videos that considered the embodied and sensory aspects of the video home tours, that is, elements that were shown to us, not just the verbal narratives. From this approach, the codes were categorised into themes, and then into conceptual framings to explain the narratives being shared.^40^ The transcripts from the audio tours were analysed by reading and rereading the texts to see if the same coding categories and themes emerged as with the video data, and to explore any alternative findings. The development of the following three themes was created from this iterative process, namely: (i) Connected home; (ii) Practices of home; and (iii) Displaying family and home. We will now develop each of these themes further.

## 3 FINDINGS

### 3.1 Theme 1: Connected home

**FIGURE 1:**
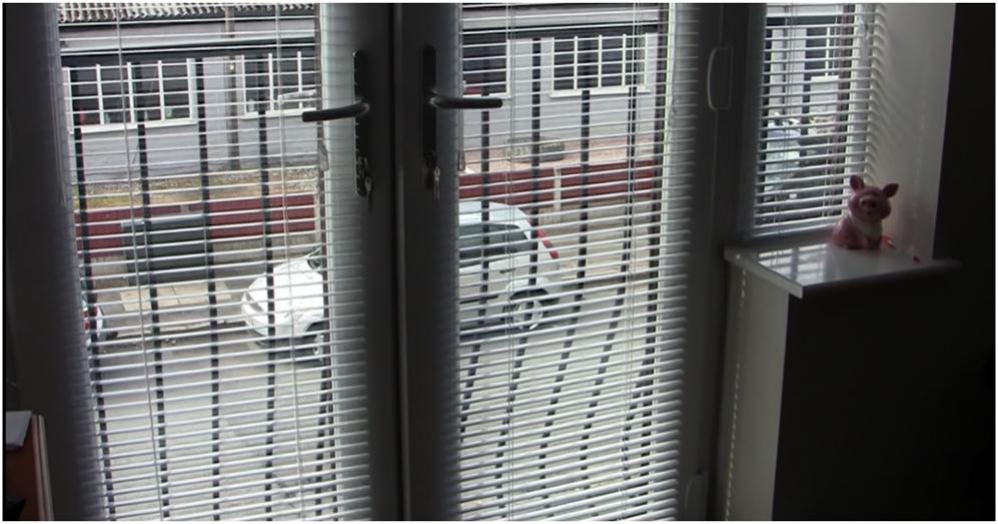
Lily's window view towards the gym opposite.

The data revealed that a person living with dementia's home is an interconnected part of the neighbourhood and a starting point for understanding their neighbourhood. Connections between home and neighbourhood were seen to be fluid and to flow out of and into the home. For example, as seen in Figure 1, Lily described the vital viewpoint her windows gave her onto the street below where she was privy to the comings and goings in the neighbourhood.

Beyond Lily's apartment was a low‐rise warehouse that housed a gym. Lily shared how predominantly younger people were forever coming and going:

I never get fed up just sitting here …you see young women you know in their leotards and that, quite happily going in and going round there…I just wish I was as young and fit as what they are. It can be busy from 7 o’clock…see those two tires I woke up one morning and a young man and a woman were bouncing a mallet off each one so I opened the veranda door and I said, ‘excuse me I don’t have to be up at quarter to 7′ and so they said they were sorry and they didn’t realise we could hear it in this building and they don’t do it now until after 8 o’clock. I don’t care if they do it all day after 8 o’clock.

The moment described by Lily contains a sense of connection between indoor and outdoor spaces of the neighbourhood and an interaction between the two.

The boundaries between home and neighbourhood were seen to be dynamic and flexible and, as such, this theme reflects how participants were interconnected with their wider neighbourhoods and the natural world through the thresholds of their homes. These experiences were shared by participants as they showed and discussed views through windows, or as we moved from their interior homes into gardens, backyards and to balconies and corridors and other communal spaces.

**FIGURE 2:**
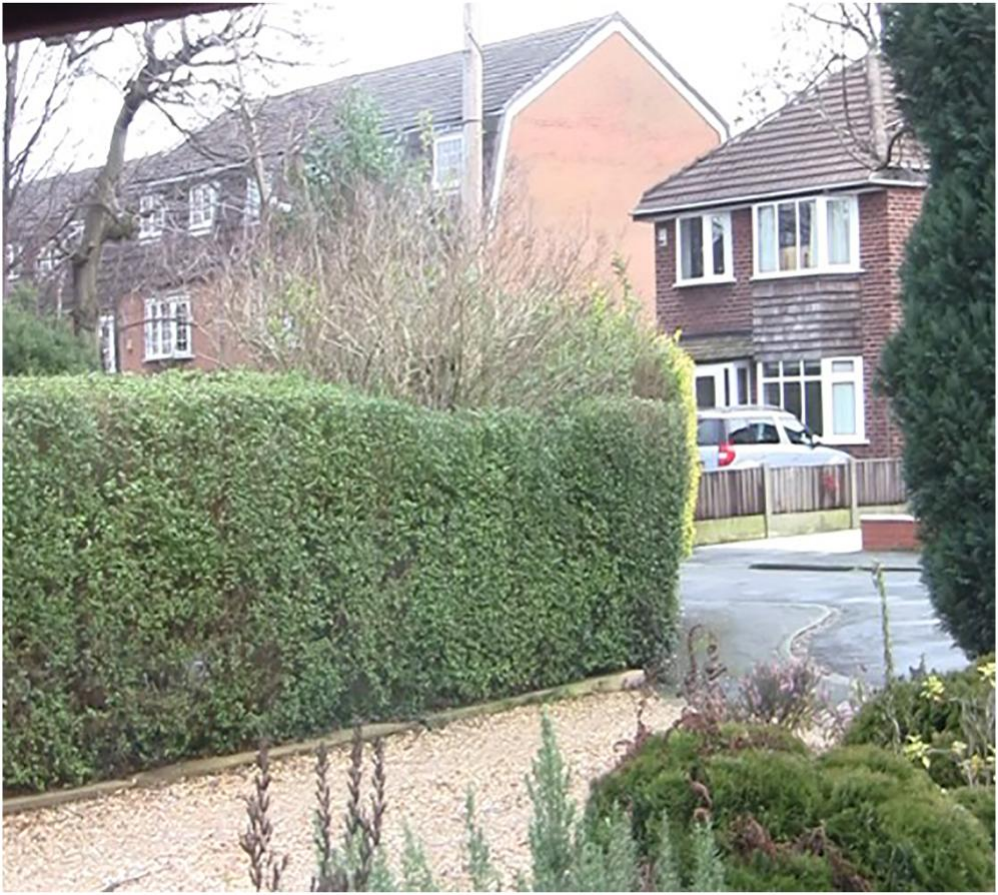
Philip's view onto his cul‐de‐sac to see the comings and goings.

Phillip had a strong sense of belonging within the cul‐de‐sac where he had lived for more than 60 years since the houses were first built—see Figure 2.

Philip knew everyone who lived there and enjoyed the view from his window‐facing seat where he could see the comings and goings of the neighbourhood, exclaiming *“I can see what's going on without being nosey, if a car comes along I can see where it's going or anything like that…we know everyone—we are all friendly to everybody”.*

Similarly, Dennis, who also lived in a cul‐de‐sac, talked about seeing who was *“driving off to work for the day”* and who was *“staying home”*. These experiences of connecting with neighbourhoods from home can be of increased importance for people living with dementia who may no longer go out as much as they once did. In some of our other work we have highlighted the feeling of insecurity and loneliness that people living with dementia have described about the ‘quietness of neighbourhoods’ when others are at out at work or other activities.^41^ These experiences enabled Phillip and Dennis to remain a part of the daily rhythms of their neighbourhood from the inside of their homes and, as such, to feel a sense of connection through their interior viewpoints. Many participants described the importance of window views to support encountering everyday life outside.^42^ These observations and interactions through window views disrupted the notion that home is ‘private’ and the street ‘public’; instead, there is a fluidity between the boundary of home and street that enables ongoing interactions with neighbourhood life.

However, not all experiences were positive. Jean described her deteriorating relationship with the place she had lived since childhood, partly due to the increasing challenge of litter, dog dirt, and transient neighbour population. Unlike most participants who arranged their seating to provide a view of outside, Jean positioned her whole body facing away from her front window stating *“so I can't see what's going on outside”*. She also showed her closed window blind, which had not been opened for more than 2 years, in order to physically and metaphorically ‘shut out’ her neighbour's front yard which was overflowing with rubbish.

Neighbourhoods do not remain the same and these experiences highlight the complexity of ageing in place. Jean no longer felt the same sense of belonging to a place that had previously embodied a strong sense of identity. The interior of Jean's home became an important place of resistance from ‘the outside’ as she continued to maintain a sense of identity inside her home.^43^ Moreover, participants regularly reported the enjoyment they experienced from being connected to the natural world through their windows and to the sensory experience of the seasons coming into their homes.^42^

**FIGURE 3:**
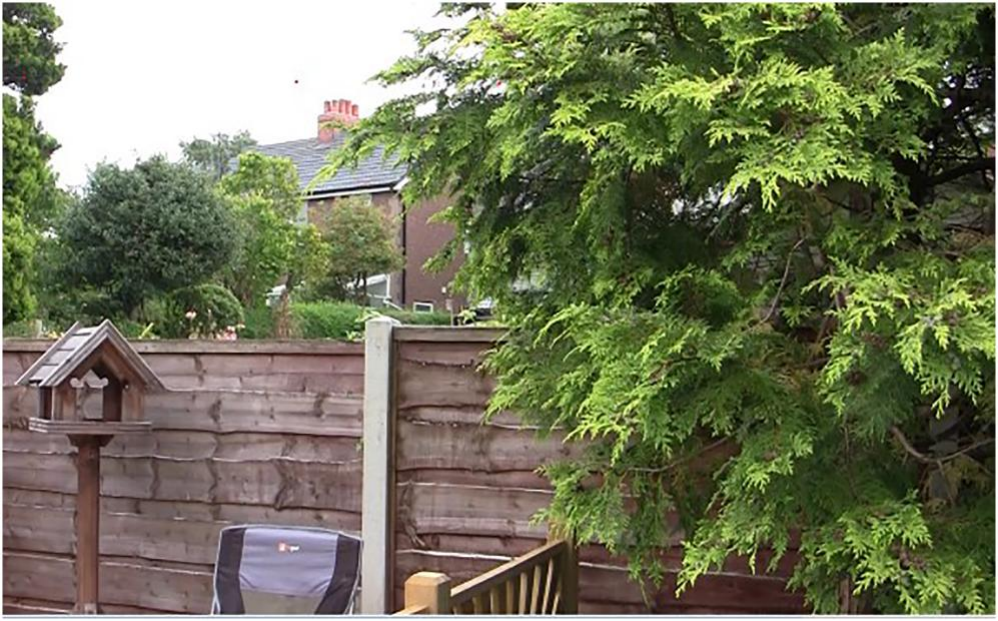
Dennis' garden and neighbourhood view.

The importance of the multi‐sensory experiences of the natural world for people living with dementia has been cited as holding value for mental health and wellbeing.^44^ The experience of remaining connected to the beauty of the natural world was mood‐enhancing. Peggy commented on liking her *‘wee side window’* because it brought more brightness into the flat. From here she could see the distant hilltops and she pointed out the snow that remained on them and described the trees as just getting their leaves back after being bare through winter. Noting the passing of seasonal time, Lily too described the importance of the brightness for lifting her mood in her small apartment, and Amobi *‘chased the sun’* that filled different parts of his house from morning to afternoon via a collection of carefully positioned chairs. As seen in Figure 3, gardens and backyards were important spaces for both social neighbourhood connections and with the natural world.

In sum then, windows, balconies, gardens and other outdoor spaces provided opportunities for social connection by seeing the daily rhythms of the street and of observing, and being observed by, others who come and go. The outdoor spaces of the home also enabled a sense of connection to nature that is experienced through a range of senses. Arguably, both these points give rise to a sense of connection to other people and things beyond the home.

### 3.2 Theme 2: Practices of home

This second theme reflects what participants shared about the everyday routines of home life. They showed how they continued to contribute to caring practices while also discussing some of the challenges and shifting experiences of domesticity. The routines and regular aspects of the upkeep of everyday domestic life that were discussed by participants ranged from shopping, cleaning, cooking, gardening and on to activities such as laundering. As Isobel showed us around her home she said, *“I want a nice home…I want a clean home”* and, as if to demonstrate as we moved through her home, she tended to the things she noticed, such as pruning a houseplant's dead leaf and removing an out‐of‐place cleaning product.

**FIGURE 4:**
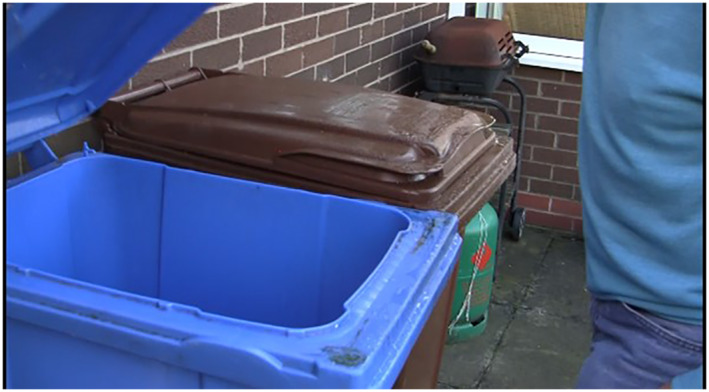
Dylan's recycling containers.

Other participants also noted out‐of‐place things or jobs that needed to be tackled as we moved through their homes, perhaps conscious of the filmmaking and concerned that untidiness and/or incomplete tasks would be viewed as a comment on their competency in living with dementia as the following examples indicate:

- Peggy, who had not yet put her shopping away, did not want it captured on film.
- Sal, who heard her tumble dryer finish whilst we were filming and, alerted by the familiar sound of the cycle ending, immediately bent down to begin unloading the clothes. She told the researcher (SC) that she spent much of the day cleaning and demonstrated this by wiping her kitchen sides and laughing.
- Dylan, who described activities that he was responsible for in the home. He said his wife did the cooking, but he did the cleaning and hoovering every Saturday, and the weekly recycling. With some pride, he took SC outside to show all the recycling bins explaining what each of the various coloured bins were for and what went in each container, a practice shown in Figure 4.

Not all participants remained as engaged in the practices of home as they once had been. Some had formal support with carers coming to help with bathing and medications. Thomas, who had once been very handy at home, challenged the narrative of what he could no longer do by laughingly saying *“I still do the pots though don't I?”*. However, Thomas' words contained a deep sense of sadness and this moment was captured on film in the look that passed between Thomas and his wife. Occasionally, there were unspoken moments like this visible on film, where there was silence, laughter, or a glance that passed between participants and their spouses. These intimate moments and glances highlighted some of the challenges that were synonymous with the loss of relational roles and expectations.^10^

**FIGURE 5:**
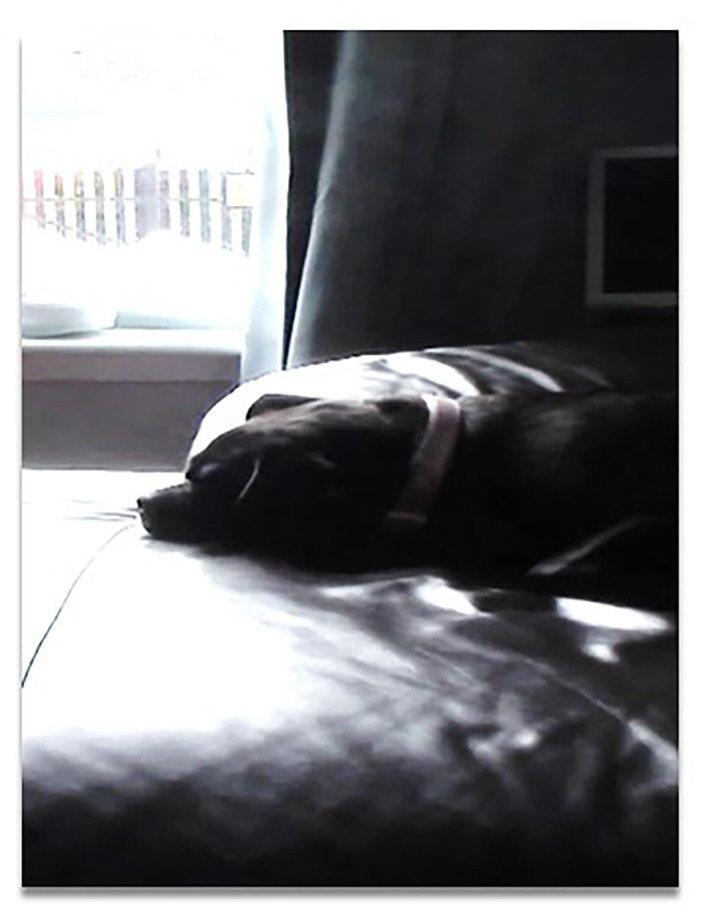
Murry's much‐loved dog.

Home also continued as a site for practices of caring for others. This was revealed in the spaces of the home that housed toys for visiting grandchildren to play with and spare bedrooms decorated and ready for overnight visits. Notably, several participants owned dogs or cats and sometimes they accompanied participants on the home tour, following us around as ever‐present family members. As shown in Figure 5, Murray's dog was a constant companion.

Companionship was also attributed to Anna's cat and Suzanne played an active role as the main dog walker in the household. Dogs, cats, other pets and visiting grandchildren all illustrated how people living with dementia continued to provide a caring role within their homes. Despite narratives of loss associated with dementia, participants reframed dementia through their ability to continue to engage in caring roles and everyday routines of home.^45^

### 3.3 Theme 3: Displaying home and family

Home was revealed not only as a place for activity or ‘doing things’ but also in how the intimacies of home and family were ‘displayed’.^9^, ^46^ For many participants, family, and other relational connections, were on display throughout their homes via photographs depicting family members that revealed generations of family connections. At times, favourite and treasured objects took the attention of participants that prompted shared stories rooted in biographical history. Home tours provided participants with the opportunity to engage in narrative citizenship and share their own stories supported by displays of home and family and the memories they held.^47^

**FIGURE 6:**
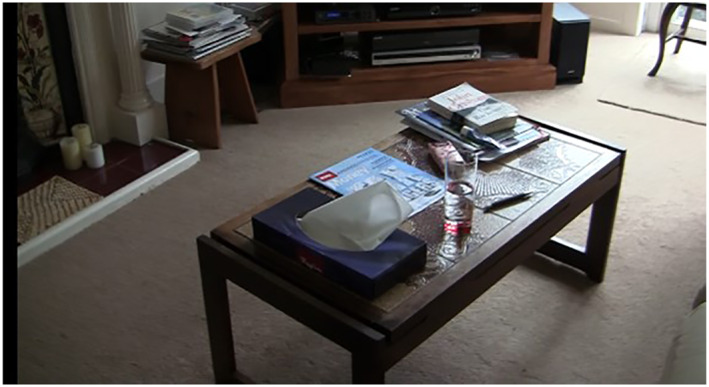
Suzanne's sentimental coffee table.

Photographs of important and notable individuals and life events, such as graduations, weddings and special gatherings, displayed narratives about relationships and belonging within a family and wider network. Sometimes photographs went beyond being merely a material object. For example,:

- Peggy, who lived alone, described her many photographs as “*like …company…I treat them* [she points to the photographs] *as if they're* [the relatives in the photographs] *here”*. They materialised a presence for Peggy of her family, keeping her company and preventing her from feeling so alone in her apartment.
- Grace, showed an old Bible as a treasured possession that was always kept on her bedside table. During the home tour, Grace picked up the Bible and, flicking through its pages, said, *“this is my mother's Bible”.* The Bible provided an important link to her spiritual identity and embodied a precious connection to her mother. Material objects have significant power for enabling storytelling for people living with dementia, and this can be supported through the holding and touching of objects.^48^
- Amobi, prompted by his wife, shared a wooden comb from his country of birth and which held particular significant connections to this other important place of belonging and identity.
- Suzanne, shared that the coffee table, which was a central feature in her living room, held important sentimental value as it represented a significant transitional moment in creating a home. The coffee table, shown in Figure 6, had been studiously saved for and purchased before she and her husband had married, having eventually become affordable in a shop sale. As Suzanne emphasised *“we'll never, ever, ever let go of it!”*.

These mundane objects were ‘vital’ holding significant affect for participants^49^ and displayed the relational, embodied nature of home. The objects were also significant in supporting a sense of identity and meaning for participants through a narrative citizenship that emerged during the home tours and sharing objects that held symbolic meaning.^47^ Whilst participants did not always remember the objects immediately, through their handling and holding memories were recalled. A good example of this recollection was when Roger held, and turned in his hands, the cricket bat that had valued autographs on its face. At this moment of object touch, the memories of past times came flooding back as Roger recollected past cricketers, *“He was possibly the greatest Australian—Don Bradman and these are England from 1946‐47 team”*.

Sometimes participants wanted to avoid having aspects of their homes filmed, this included not wanting to share messy spaces and areas that were displaying the ongoing practices of domestic life such as drying laundry. This avoidance sometimes related to not wanting to share spaces and objects on display that represented the loss of previous competencies and abilities, which suggests that display is a complex phenomenon. Suzanne wanted to avoid a focus on a keyboard which she no longer played, but she did choose to show the researcher (SC) a pile of clothes lying on her bedroom floor, which she qualified with saying that getting dressed in the morning was “*one of the most challenging aspects of my day”*. Suzanne then went on to reveal that she *“struggled to organise and put away items of clothing”* and that now *“my husband has to sort out my clothes”.* The pile of clothes therefore remained lying on the bedroom floor until this task was completed.

The home tours also revealed the fluidity of family life. Home was not a static place, but one that endured changes within families that included bereavement, loss, and illness. As participants showed their homes during the tour, they revealed displays of family life and how their home had been adapted to accommodate diminishing health and ageing bodies ‐ and the impact this had had on relationships. For instance, Albert and his wife had re‐purposed the upstairs spaces in their home that had once been their children's bedrooms. They no longer shared a bed together and slept in separate bedrooms suiting their current routines and displaying how marital life can often change with the onset of health conditions. Both Albert and his wife had multiple illnesses that impacted their physical comfort and ability to sleep. This was also true for Phillip and his wife who no longer shared a bed. Whilst it is known in the literature that sleep issues become more prevalent in older age,^50^ many carers in the study complained of sleep disturbances and to cope with the resultant exhaustion^51^ it appeared that couples decided to sleep apart. The home tours displayed these changes through the intimacies of partnered life enabling us to be privy to these private spaces.

**FIGURE 7:**
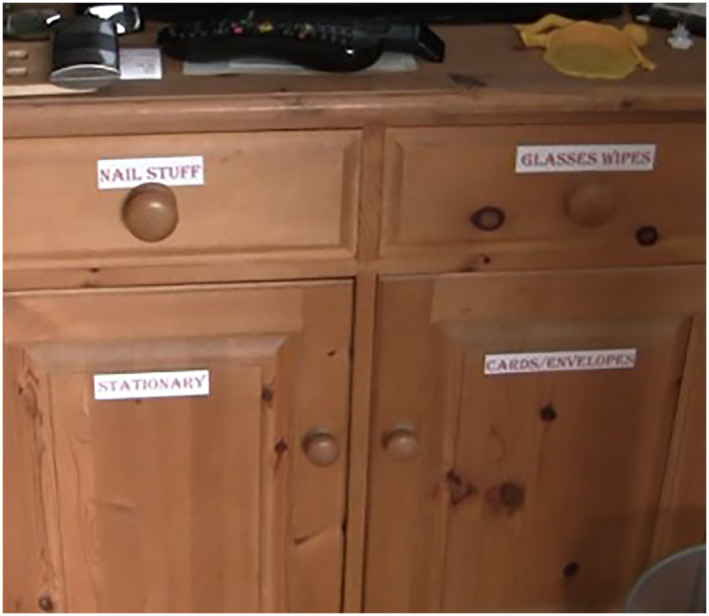
Susan's cupboards with labels showing what is kept inside.

Dementia was also displayed through changes to the material home as participants shared how they coped and managed the challenges of dementia, such as memory loss, and shared individual strategies for managing forgetfulness. As Figure 7 reveals, Susan had labelled cupboards around her home stating what was kept inside, and although she said seeing the labels was uncomfortable, it enabled her more independence in finding things and in reducing her anxiety.

Sean showed a spare bedroom that was now crammed so full of his belongings that the door could no longer be fully opened. He said he was *‘a hoarder’* and noted that this was something *‘to be dealt with’*. This was a challenging display of the burden of home life, the accumulation of things, and the difficulty with coping with the processes of sorting and parting with collected items.

## 4 DISCUSSION

This paper has provided an exploration of 46 participatory home tour interviews undertaken with people living with dementia to provide further insights into what matters about home from their perspective. Indeed, the home tours revealed how ‘home’ extended beyond boundary walls into outside spaces and continued to enable connections to local spaces. Contrary to notions of home as a private space set apart from the activities of the neighbourhood, home is revealed as not always quite so private. The meaning of home is shown to have fluid boundaries and to show the importance of supporting neighbourhood connections to be maintained (where desired).

Musselwhite^42^ suggests that views from windows enable a connection with neighbourhoods, or with the outside world. Participants were able to connect with others through outside spaces, such as gardens and balconies, that were important extensions of home and were thresholds to neighbours and neighbourhood.^22^, ^34^ Outside spaces and viewing points also offered valuable opportunities to enjoy the sensory pleasures of flowers, trees, wildlife and the experience of the sunlight. For some participants, such as Philip and Dennis, their sense of belonging was supported through home being connected to their neighbourhoods, whilst for others, such as Jean, the neighbourhood needed to be physically and metaphorically shut out. It was important for participants to feel in control of the boundaries between inside and outside their home (i.e., between private and public spaces), such as where they chose to sit and whether or not this had a views from the window, and through access to outside home spaces. Such choices about window views revealed the inter‐relationship between home and neighbourhood and highlighted complexities within an ageing in place discourse.^42^

The tours show how life at home goes on for people living with dementia, as people develop creative adaptations to homes to cope with the everyday challenges of living with a condition that impacts memory, decision making and everyday functionality.^52^, ^53^ Sometimes these are small, mundane adaptations, such as memo reminders, or labels on cupboards. During the home tours, participants actively took part in practices of home as they became involved in tidying up familiar spaces as we moved through their home. Lovatt^54^ points to the value of ‘doing home’ for older people in residential care who create a sense of home by carrying out everyday practices such as cleaning and hosting. This connects the person to their previous experiences of home life. Engagement with practices of home was a vital part of continued homemaking for participants and demonstrated their ongoing competencies within the home. Nygård and Starkhammar's^55^ study on everyday technologies at home suggests that home is often a place where people living with dementia can feel free from scrutiny and surveillance. This is especially important when the outside world might prove more challenging, or highlight difficulties such as orientation in getting around or remembering how to do things. Home could, at least, be adapted, afford less scrutiny, and new coping strategies could be tested and errors overlooked.

Home is a place to uphold a sense of self that can be maintained through everyday practices and through the memories and relationships contained there. However, tensions arise, for instance with some participant's feeling that they no longer had the autonomy they had once had and where there was awareness of losses. So, home could emerge as ‘contested territory’ as couples disagreed about what the person living with dementia could still do, or how to manage challenges in the home.^21^ Sometimes, spouses contradicted what the person living with dementia was saying during the home tour. Couples had to constantly negotiate and renegotiate responsibilities in the home as the person living with dementia became unable to undertake everyday tasks—leading to frustration or loss for those involved. Our work adds to the significant work by Brannelly and Bartlett^2^ that explores the importance of valuing interdependence to ensure that support for people living with dementia to remain living at home recognises the importance of key relationships and ongoing changes to peoples' lived experiences.

Home also connected participants to family and friends through the display of objects and photographs around the home. These memories provided a sense of connection for people living with dementia in their own homes and supported a sense of identity, to the extent that Peggy considered her photographs to materialise her family and hence to support her connectedness to them. Important memories were shared through material objects on display, such as through Suzanne's coffee table and Roger's cricket bat. These displays of home^9^ enabled narrative citizenship^47^ for people living with dementia and demonstrated how embodied memories could be ignited through the handling of an object or by pointing out a great‐grandchild in a photograph.

Whilst home life did not remain static, and the impact of life with dementia had repercussions for relationships and household activities, the participatory nature of the home tours enabled people living with dementia to share important narratives about what mattered most from their perspective. In particular, the home tours revealed how people living with dementia experienced home and that the home held the imprints of lives lived in the past and present and into the future.

## 5 CONCLUSION

For individuals living with dementia, home needs to be considered as more than a functional, physical space to be adapted, or a space where care can be provided. Our study provides new insights into the ways people living with dementia continue to engage in homemaking and in the everyday activities of home. Importantly, the study shows how home can support connections to others through an interaction between home and neighbourhood. We suggest that knowing what matters to people living with dementia about their homes is a crucial element in developing policy that enables and supports people to remain part of their neighbourhoods for as long as they desire to do so. Understanding home through the interdependence of connections, roles and memories will be important in supporting decisions about care and support over time. Furthermore, it is crucial to support the rights of people living with dementia to remain living within their homes and neighbourhoods.^16^

### 5.1 Limitations

The participants in this study did not reflect the diversity of racial and ethnic backgrounds within the UK. Most participants identified as White British, and this is a limitation of the work. Participants were also mostly living within heterosexual relationships or had been previously. We did not carry out the home tours in the Swedish fieldsite and it would have been a useful comparison to understand home within a different policy context to the UK.

### 5.2 Future research

It would be helpful to understand ‘home’ for people living with dementia in different contexts, and with diverse identities and lived experiences. This research has indicated a connection between home and neighbourhood, and it would be helpful to understand more the relationship between different types of homes and the neighbourhood, including residential care homes.

## CONFLICT OF INTEREST STATEMENT

All authors declare that they have no conflict of interest.

## Data Availability

Research data are not shared.
